# Long-term survival after adrenal metastasectomy from colorectal cancer: a report of two cases

**DOI:** 10.1186/s40792-019-0611-z

**Published:** 2019-04-15

**Authors:** Akinori Tsujimoto, Takeshi Ueda, Hiroyuki Kuge, Takashi Inoue, Shinsaku Obara, Takayuki Nakamoto, Yoshiyuki Sasaki, Yasuyuki Nakamura, Fumikazu Koyama, Masayuki Sho

**Affiliations:** 1 Department of Surgery, Nara Medical University, 840 Shijo-Cho, Kashihara, Nara, 634-8522 Japan; 2 Department of Endoscopy, Nara Medical University Hospital, 840 Shijo-Cho, Kashihara, Nara, 634-8522 Japan

**Keywords:** Colorectal cancer, Adrenal metastasis, Adrenalectomy, Long-term survival

## Abstract

**Background:**

Solitary adrenal metastasis from colorectal cancer is rare. Adrenal metastasis is usually detected with synchronous multiple metastases in other organs and is, therefore, considered to be unsuitable for surgical resection. The long-term outcomes of patients with solitary adrenal metastasectomy from colorectal cancer have been reported; however, the survival advantage has not been established. We herein report two cases of curative adrenal resection in patients with solitary adrenal metastasis from colorectal cancer who achieved long-term survival of > 9 years without recurrence after surgical resection.

**Case presentation:**

The first case involved a 71-year-old man who underwent abdominoperineal rectal resection for rectal cancer. Preoperative CT revealed a mass in the right adrenal, which was growing after surgery. After chemotherapy the adrenal mass decreased in size, and adrenalectomy was performed at 8 months after the first surgery. A pathological examination confirmed metastasis from rectal cancer. The patient received adjuvant chemotherapy and is currently alive without recurrence at 9 years after the adrenalectomy. The second case involved a 53-year-old man who underwent sigmoidectomy for sigmoid colon cancer. Four years later, lobectomy was performed for isolated lung metastasis. Twenty months later, PET-CT revealed solitary metastasis in the left adrenal gland and adrenalectomy was performed. A histopathological examination revealed metastatic adenocarcinoma of sigmoid cancer. Postoperative chemotherapy was administered after adrenalectomy and the patient is currently alive and apparently disease-free at more than 9 years after undergoing adrenal metastasectomy.

**Conclusion:**

Curative resection for solitary adrenal metastasis from colorectal cancer may be beneficial for survival.

## Background

Metastasis to the adrenal gland is a relatively frequent autopsy finding in cancer patients. Adrenal metastasis from colorectal cancer (CRC) is not rare; however, it usually occurs in patients with multiple synchronous metastases in the terminal stage of cancer. Solitary and clinically curable adrenal metastasis from CRC is extremely unusual. In cases involving pulmonary or liver metastases from CRC, successful resection procedures have a clear survival benefit (27–37% 5-year survival in the liver, and 32–40% in the lung) [1–4]; however, the significance of solitary adrenal metastasectomy is unknown. Several studies have reported a favorable prognosis and notable benefit in selected patients following surgical resection for adrenal metastasis [5–11]. We herein report two cases of adrenal metastasectomy from CRC in which long-term survival was achieved after surgical resection.

## Case presentation

### Case 1

**Fig. 1:**
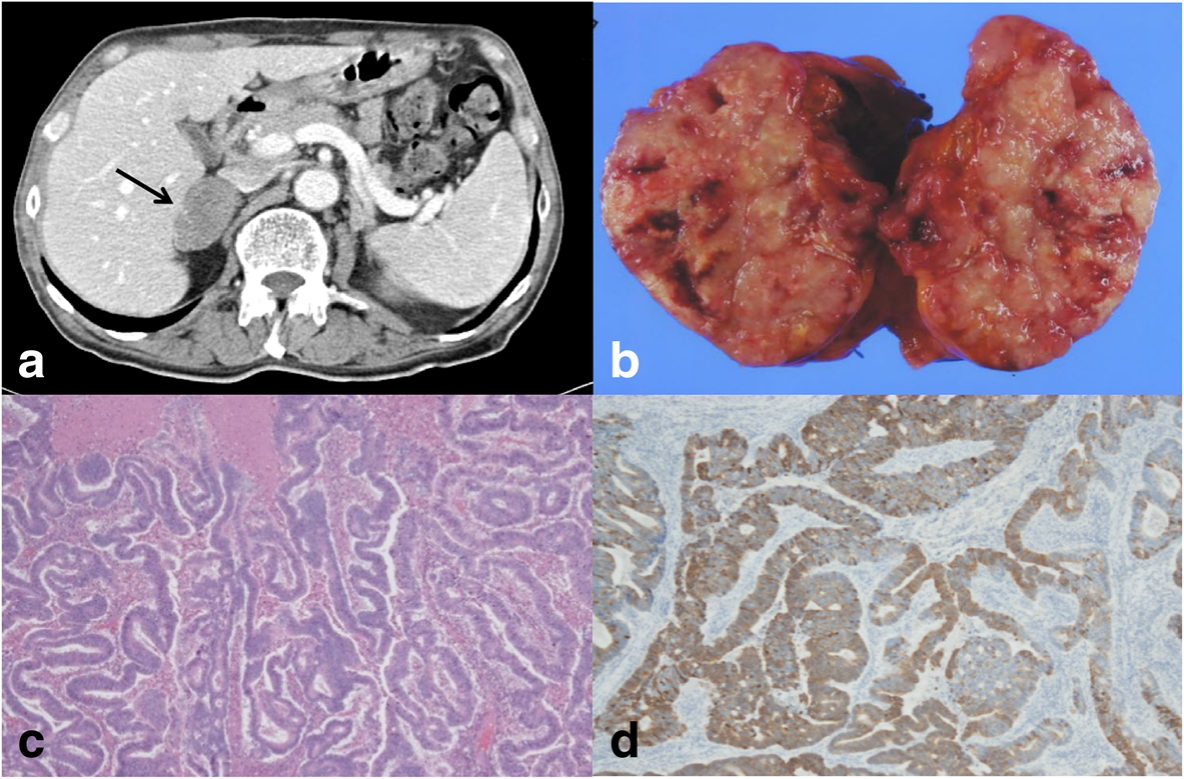
The clinicopathological findings in Case 1. **a** The preoperative findings: Abdominal CT showed a right adrenal tumor 4.7 cm in size (black arrow). **b** The macroscopic appearance: A cross-section of the adrenal tumor was solid and grayish white. **c** The histopathological findings: The histopathological examination of the adrenal tumor showed adenocarcinoma, which was compatible with metastasis of primary rectal cancer. **d** The immunohistochemical findings (CK20 staining): Adrenal tumor cells were positive for cytokeratin20 and negative for cytokeratin7.

**Fig. 2:**
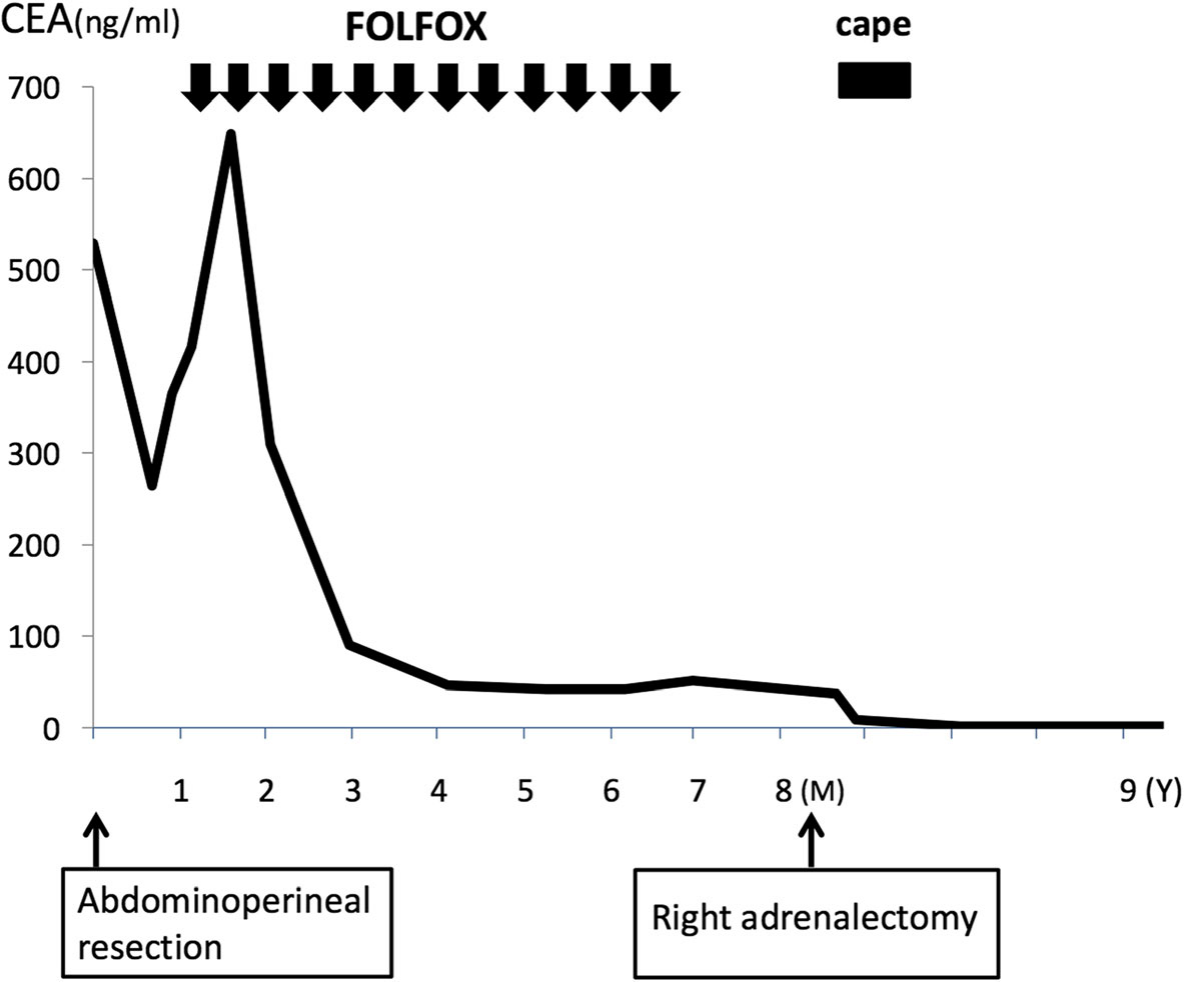
The clinical course of Case 1. Vertical axis: CEA (ng/ml). Horizontal axis: time elapsed. M month(s). Y year(s). FOLFOX infusional 5-fluorouracil, leucovorin, oxaliplatin. Cape capecitabine

A 71-year-old man who presented with anemia was diagnosed with rectal cancer with a synchronous adrenal metastasis. His serum level of carcinoembryonic antigen (CEA) was 529.8 ng/ml; and other laboratory data showed no abnormalities. Preoperative abdominal computed tomography (CT) revealed a mass in the right adrenal gland of 4.3 × 3.2 cm in size (Fig. 1). Abdominoperineal rectal resection with regional lymph node dissection was performed. The pathological findings revealed well-differentiated adenocarcinoma. According to the TNM classification, the disease was stage IV (fT3N1M1[ADR]). The metastatic adrenal lesion increased in size and the CEA level became elevated after the operation (Fig. 2). After chemotherapy with 12 cycles of FOLFOX, the adrenal mass shrunk in size, the CEA level markedly decreased, and no new lesions were detected (Fig. 2). On admission, the results of general blood tests including the adrenal hormone levels were normal. Right adrenalectomy was performed for 8 months after the first surgery. The pathological examination of the adrenal gland confirmed the diagnosis of adenocarcinoma, and was consistent with metastatic rectal cancer (Fig. 1). The CEA level normalized after the right adrenal resection. Capecitabin was administered as post-operative chemotherapy for 9 months after adrenalectomy. Thereafter, the patient was closely followed without therapy. The patient is currently alive at 9 years after adrenalectomy with no evidence of metastasis and a normal CEA level.

### Case 2

**Fig. 3:**
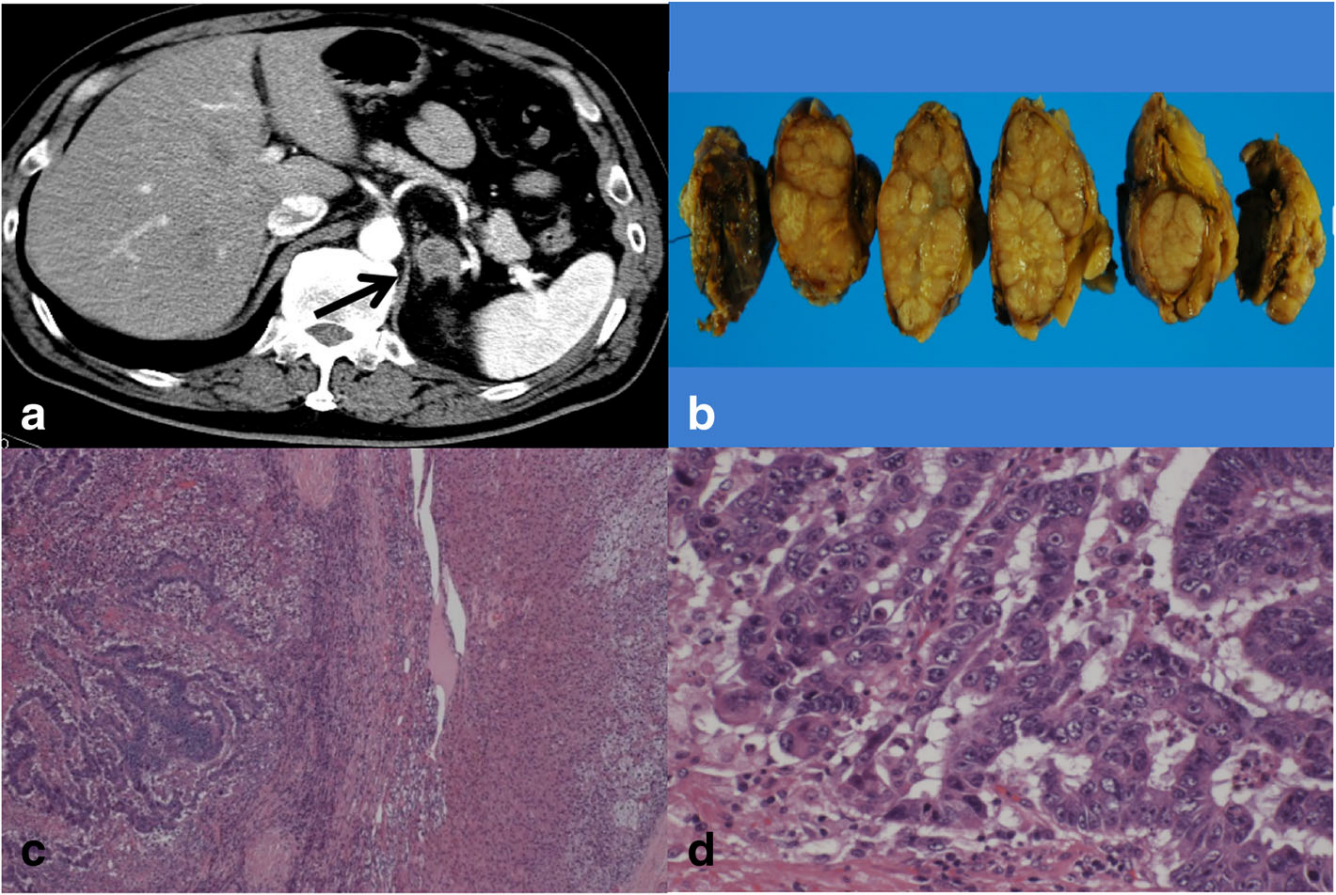
The clinicopathological findings of Case 2. **a** The preoperative findings: Abdominal CT showed an enlarged left adrenal gland (black arrow). **b** The macroscopic appearance: The cross-section of the adrenal tumor was solid and grayish white. **c**, **d** The histopathological findings: The histopathological examination of the adrenal tumor showed adenocarcinoma, which was compatible with metastasis of primary colon cancer

**Fig. 4:**
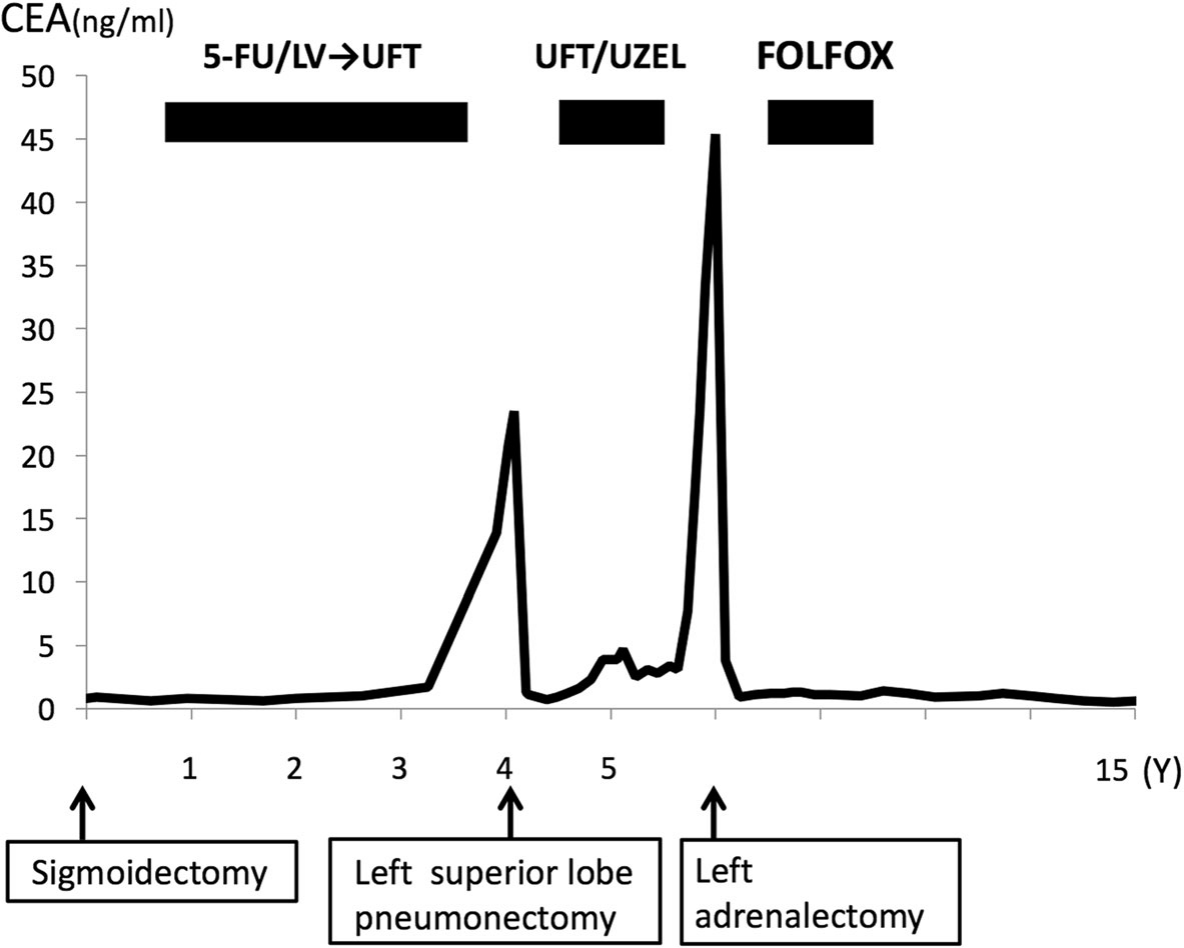
The clinical course of Case 2. Vertical axis: CEA(ng/ml). Horizontal axis: time elapsed. M month(s). Y year(s). 5-FU 5-fluorouracil LV leucovorin. UFT tegafur-uracil. UZEL calcium folinate. FOLFOX infusional 5-fluorouracil, leucovorin, oxaliplatin

A 53-year-old man underwent sigmoidectomy for sigmoid colon cancer. A pathological examination showed poorly-differentiated adenocarcinoma. According to the TNM classification, the disease was stage IIIb (fT3N2M0). Preoperative laboratory analyses, including the serum level of CEA (0.8 ng/ml), showed no abnormalities. After surgery, chemotherapy with 5-fluorouracil and folinic acid followed by tegafur uracil were administered. At 46 months after the operation, the patient’s CEA level increased to 13.9 ng/ml and positron emission tomography (PET)-CT showed an abnormal uptake in the upper lobe of left lung, and left upper lobectomy was performed. The pathological examination revealed metastatic adenocarcinoma from the previously resected sigmoid colon cancer. Oral adjuvant chemotherapy with tegafur uracil and calcium folinate was administered after lobectomy. Although the patient’s serum CEA level normalized after surgery, it increased to 23.3 ng/ml at 20 months after lobectomy, and PET-CT revealed a left adrenal metastasis (Fig. 3). No additional recurrence was observed on CT or colonoscopy. On admission, the results of general blood tests, including the patient’s adrenal hormones levels, were normal. Left adrenalectomy was conducted nearly 6 years after the first surgery. The histopathological findings showed metastatic adenocarcinoma of sigmoid colon cancer (Fig. 3). Additional chemotherapy with FOLFOX was administered for 12 cycles after adrenalectomy. At the time of the most recent follow-up examination (9 years after the last chemotherapy treatment following resection of adrenal metastasis), no recurrence was detected by CT or colonoscopy and the patient’s CEA level was normal (Fig. 4).

## Discussion

Adrenal metastasis is a relatively common autopsy finding in cancer patients. The types of cancer that most frequently metastasize to the adrenal gland include lung cancer, breast cancer, and renal cell carcinoma [12–15]. The incidence of adrenal metastasis from CRC ranges from 3.1 to 14.4% [12–16]. However, metastatic adrenal disease is often detected in the presence of multiple synchronous metastases at other organs during the terminal stages of cancer, whereas solitary adrenal metastasis is rarely identified. During the diagnostic work-up of CRC patients, although the incidence of adrenal incidentalomas in the patients was 10.5%, the frequency of adrenal metastases was 0.4% with widespread systemic disease, while no solitary adrenal metastases were observed [17]. Surgical resection is therefore not generally considered a viable treatment for adrenal metastasis.

Regarding adrenalectomy, Vazquez et al. reported on 166 patients who underwent adrenalectomy for adrenal metastasis, including CRC, and found that an aggressive surgical approach improved the overall survival in patients with metastasis from tumrors of the soft tissue, kidney, lung, pancreas, and other areas [18]. Muth et al. reported 30 patients undergoing surgery for adrenal metastasis and found that the prognosis for patients with metastasis from CRC was better than that for other malignancies [19]. In addition, several case reports [5–11, 23] have suggested that resection of solitary adrenal metastasis from CRC might improve the prognosis. However, Kim et al. reported that the 5-year survival of 37 patients who had undergone adrenalectomy for metastatic disease was 24% (median, 21 months), and a disease-free interval exceeding 6 months and complete resection were the only predictors of an improved survival [20]. This means that a prolonged survival can be achieved in select patients, but the benefits of adrenalectomy remain controversial. In our first case, the patient with synchronous adrenal metastasis from rectal cancer had first resected primary lesion because of anemia and bleeding from cancer. After completing chemotherapy, adrenalectomy was planed after first confirming that no systemic metastasis other than that in the adrenal gland was observed. In the second case, metachronous solitary lung and adrenal metastasis from colon cancer roughly 4 and 6 years after the first operation, respectively, were resected upon recurrence. Both cases were solitary or oligometastasis without systemic metastasis and completely resectable, which may have led to the long-term survival exceeding 9 years after adrenalectomy.

The early detection of solitary adrenal metastasis may offer a chance to achieve curative resection and a good prognosis. It is important to consider the possibility of adrenal metastasis from CRC during follow-up after primary surgery. However, patients with adrenal metastasis are generally asymptomatic, without abdominal pain or adrenal insufficiency, as was observed in the present cases. Previous reports showed that the serum CEA level is a useful indicator of the presence of the adrenal metastasis [21–23], which can be confirmed by the imaging modalities such as CT, magnetic resonance imaging (MRI), and PET-CT. In most cases, including ours, the CEA level was elevated when adrenal metastasis was detected and decreased considerably after adrenalectomy. The CT findings in our patients showed that the adrenal gland had a heterogeneous low density, which has previously been reported [24, 25]. MRI is also effective for distinguishing adenomas, carcinomas, and metastases in cases of adrenal incidentaloma [25, 26]. PET-CT could differentiate between benign and malignant adrenal lesions, with 93–100% sensitivity, 80–100% specificity, and 92–100% accuracy [27]. In our case, PET-CT was useful for detecting adrenal metastasis and excluding metastatic disease elsewhere prior to a potentially curative adrenal resection [27, 28]. Cedermark et al. reported that metastasis to the lungs is associated with a higher incidence of adrenal metastasis [16]. In the second case, adrenal metastasis was detected 20 months after performing lobectomy and 6 years after the initial surgery. This suggests hematogenous metastasis from the primary lesion via the lung to the systemic venous circulation and adrenal gland. Thus, long-term follow-up (more than 5 years) after resection of CRC might be important for the early detection of adrenal metastasis, especially after lung metastasectomy.

The performance of metastasectomy for common cancer types has substantially increased [29]. It is generally accepted that solitary metastasis should be resected to achieve a good prognosis; however, the incidence of truly resectable lesions is low. Patients with stage IV CRC have a very poor prognosis, with a 5-year survival rate of only 10–20%. Shimomura et al. reported the survival benefit of metastasectomy in patients with stage IV CRC and noted that the 5-year overall survival rate of patients who underwent curative resection (R0) was 45.9%, while the rates of patients who underwent non-curative resection (R1, 2) were 12.5% and 6.7%, respectively [30]. They noted that the T stage (T4), histological type (other than well-differentiated adenocarcinoma), an elevated serum CEA level (≥ 30), and the presence of extra hepatic disease were prognostic factors for the curable group, whereas only the presence of postoperative chemotherapy was a prognostic factor for the non-curable group. Although the overall survival of patients with curable resection was significantly better than that of those who underwent non-curable resection (*p* < 0.001), the survival of the high-risk sub-group (three or more prognostic factors) wihtin the curable group was as poor as that of the non-curable group. A review of reports concerning adrenal metastasis showed that patients with suspected adrenal metastasis should be considered candidates for adrenalectomy when: (1) control of extra-adrenal disease can be accomplished; (2) metastasis is isolated to the adrenal gland(s), adrenal imaging is highly suggestive of metastasis, or the patient has a biopsy-proven adrenal malignancy; (d) metastasis to the adrenal gland is confirmed by a recent imaging study; and (e) the patient’s performance status warrants an aggressive approach [31]. In the present cases, both patients were low-risk and fullfilled these conditions. Thus, the operative removal of adrenal metastasis may have a survival benefit in selected patients with metastasic adrenal disease.

Based on our cases and previous reports, curative resection of adrenal metastasis from CRC might lead to a good prognosis [32–35].

## Conclusion

These cases suggested that curative resection may be effective for adrenal metastasis from CRC. The further accumulation of similar cases will be needed to confirm these findings.

## Abbreviations

CRC: Colorectal cancer
CEA: Carcinoembryonic antigen
CT: Computed tomography
TNM classification: Tumor-node-metastasis classification
FOLFOX: Infusional 5-fluorouracil, leucovorin, oxaliplatin
PET: Positron emission tomography
MRI: Magnetic resonance imaging
